# Characterisation of the Novel Mixed Mu-NOP Peptide Ligand Dermorphin-N/OFQ (DeNo)

**DOI:** 10.1371/journal.pone.0156897

**Published:** 2016-06-07

**Authors:** Mark F. Bird, Maria Camilla Cerlesi, Mark Brown, Davide Malfacini, Vanessa Vezzi, Paola Molinari, Laura Micheli, Lorenzo Di Cesare Mannelli, Carla Ghelardini, Remo Guerrini, Girolamo Calò, David G. Lambert

**Affiliations:** 1 Department of Cardiovascular Sciences, University of Leicester, Division of Anaesthesia, Critical Care and Pain Management, Leicester Royal Infirmary, Leicester, LE2 7LX, United Kingdom; 2 Department of Medical Sciences, Section of Pharmacology and National Institute of Neuroscience, University of Ferrara, Ferrara, Italy; 3 Department of Pharmacology, Istituto Superiore di Sanità, Rome, 00161, Italy; 4 Department of Neuroscience, Psychology, Drug Research and Child Health—Neurofarba, Pharmacology and Toxicology Section, University of Florence, Florence, Italy; 5 Department of Chemical and Pharmaceutical Sciences and LTTA, University of Ferrara, Ferrara, Italy; Cleveland Clinic Lerner Research Institute, UNITED STATES

## Abstract

**Introduction:**

Opioid receptors are currently classified as Mu (μ), Delta (δ), Kappa (κ) plus the opioid related nociceptin/orphanin FQ (N/OFQ) peptide receptor (NOP). Despite compelling evidence for interactions and benefits of targeting more than one receptor type in producing analgesia, clinical ligands are Mu agonists. In this study we have designed a Mu-NOP agonist named DeNo. The Mu agonist component is provided by dermorphin, a peptide isolated from the skin of Phyllomedusa frogs and the NOP component by the endogenous agonist N/OFQ.

**Methods:**

We have assessed receptor binding profile of DeNo and compared with dermorphin and N/OFQ. In a series of functional screens we have assessed the ability to (i) increase Ca^2+^ in cells coexpressing recombinant receptors and a the chimeric protein Gα_qi5_, (ii) stimulate the binding of GTPγ[^35^S], (iii) inhibit cAMP formation, (iv) activate MAPKinase, (v) stimulate receptor-G protein and arrestin interaction using BRET, (vi) electrically stimulated guinea pig ileum (gpI) assay and (vii) ability to produce analgesia via the intrathecal route in rats.

**Results:**

DeNo bound to Mu (pK_i_; 9.55) and NOP (pK_i_; 10.22) and with reasonable selectivity. This translated to increased Ca^2+^ in Gα_qi5_ expressing cells (pEC_50_ Mu 7.17; NOP 9.69), increased binding of GTPγ[^35^S] (pEC_50_ Mu 7.70; NOP 9.50) and receptor-G protein interaction in BRET (pEC_50_ Mu 8.01; NOP 9.02). cAMP formation was inhibited and arrestin was activated (pEC_50_ Mu 6.36; NOP 8.19). For MAPK DeNo activated p38 and ERK1/2 at Mu but only ERK1/2 at NOP. In the gpI DeNO inhibited electrically-evoked contractions (pEC_50_ 8.63) that was sensitive to both Mu and NOP antagonists. DeNo was antinociceptive in rats.

**Conclusion:**

Collectively these data validate the strategy used to create a novel bivalent Mu-NOP peptide agonist by combining dermorphin (Mu) and N/OFQ (NOP). This molecule behaves essentially as the parent compounds in vitro. In the antonocicoeptive assays employed in this study DeNo displays only weak antinociceptive properties.

## Introduction

While the majority of clinical opioids mainly target the Mu (μ) receptor, work in cell and animal models would suggest targeting two or more opioid receptors simultaneously may produce drugs with reduced harmful effects. A large body of this work has concentrated on simultaneous targeting of the Delta (δ) receptor to produce drugs with attenuated tolerance profiles [1–3]. Work in *in vitro* cell based systems has demonstrated that antagonism of Delta, or disruption of the Mu-Delta heterodimer, leads to recycling of the Mu receptor, rather than ubiquitination after activation and internalisation [4–6]. These findings were confirmed in animal studies with Delta knockout mice showing a complete attenuation of morphine tolerance, as did preproenkephalin knockout mice [7]. Despite this compelling evidence no Mu-Delta mixed ligands have yet reached the clinic.

The nociceptin/orphanin FQ (N/OFQ) peptide receptor (NOP) is a comparatively new member of the opioid family and is often referred to as a ‘non-opioid branch” due to little or no affinity for the non-selective opioid antagonist, naloxone [8]. The NOP receptor is located throughout the pain pathways producing anti-opioid effects supraspinally and analgesic effects spinally [8–11]. NOP has been shown to co-localise in the pain pathways with Mu [9]. Activation of the NOP receptor has demonstrated several advantages over the classical opioid receptors. For instance, NOP agonists are able to efficiently treat neuropathic pain, a condition which classical opioid do not adequately treat [8, 9]. Of particular note, intrathecal co-administration of N/OFQ and morphine in non-human primates led to a potentiation of morphine-induced antinociception, without the associated morphine-induced side effects (itch) [12]. From a cellular perspective, Mu and NOP have been demonstrated to co-express in close proximity and display differential signalling activity *in vitro*, suggesting the formation of a heterodimer [13–15].

**Fig 1:**
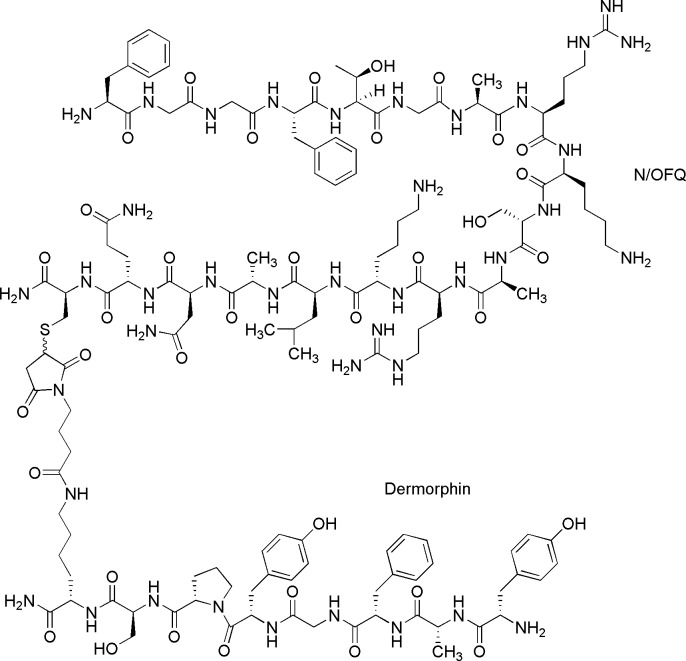
Chemical structure of DeNo.

Results from both *in vitro* and *in vivo* models have subsequently led to the development of a number of mixed molecules targeted to Mu and NOP. One of the first examples of a mixed NOP-opioid ligand was [Dmt^1^]N/OFQ(1–13)-NH_2_, a non-selective opioid agonist acting at both the classical opioid receptors and NOP. [Dmt^1^]N/OFQ(1–13)-NH_2_ demonstrated potent and sustained activity *in vivo* in the monkey tail withdraw assay, with a 30-fold increase in potency over N/OFQ [16]. The most recently developed Mu-NOP bifunctional pharmacophore is cebranopadol. Cebranopadol is a full Mu agonist and a high efficacy partial agonist at NOP [17]. In rat models, cebranopadol demonstrated a long duration of action and did not disrupt motor coordination or respiration. Furthermore, cebranopadol produced tolerance by 26 days compared to only 11 days for morphine [17, 18]. The results demonstrated by cebranopadol would suggest that targeting of Mu and NOP could provide substantial improvement in the treatment of pain. To further understand the interactions between Mu and NOP, a detailed examination of the interactions of a full agonist dual-targeted drug needs to be further made. In order to further explore the interactions between Mu and NOP we have synthesized a mixed Mu-NOP agonist named DeNo (Fig 1). The Mu agonist component is provided by dermorphin, a peptide isolated from the skin of *Phyllomedusa* frogs [19]. The NOP component is the endogenous agonist N/OFQ. We have assessed receptor binding, upstream and downstream signalling in cells and tissues and assessed in vivo spinal anti-nociceptive effects in rats.

## Methods

### Materials

The reference molecules, dermorphin, Leu-enkephalin, Dynorphin-A and N/OFQ were synthesised in house (Department of Chemical and Pharmaceutical Sciences, University of Ferrara). Tritiated UFP-101 ([^3^H]-UFP-101) was synthesized as described previously [20]. Tritiated diprenorphine ([^3^H]-DPN) and [^3^H]-cyclic Adenosine monophosphate (cAMP) were purchased from Perkin Elmer. Naloxone was purchased from Sigma-Aldrich Co. (Dorset, U.K.). Naltrindole (Delta antagonist) and [D-Pen^2^, D-Pen^5^]-enkephalin (Delta agonist, DPDPE) were purchased from Tocris (Abingdon, UK). Antibodies and protein ladders were purchased from Cell Signalling (Boston, MA, USA). All tissue culture media and supplements were obtained from Invitrogen (Paisley, U.K.).

### Synthesis of DeNo

DeNo was assembled using a classical thiol-Michael reaction; reacting in solution a thiol function inserted in the N/OFQ sequence with a maleimide function inserted into the dermorphin sequence. In detail, [Cys^18^]N/OFQ-NH_2_ was synthesized and purified in house while the synthesis of dermorphin elongated in the C-terminal with Lys(maleimide) (Fig 1) was performed using classical solid phase peptide synthesis techniques [21]. Selective deprotection of the Lys side chain of the intermediate [Lys(Dde)^8^]dermorphin-resin was achieved following the procedure of Bycroft et al [22]. To a suspension of protected [Lys(Dde)^8^] Der-resin (300 mg) in anhydrous tetrahydrofuran (3 ml), 2% hydrazine in methanol (5 ml) was added. The reaction mixture was stirred under argon for 30 min, then filtered and the resin washed 3 times with dimethylformamide (DMF,5 ml) and CH_2_Cl_2_ (5 ml). To a stirred solution of 4-(2,5-dioxo-2,5-dihydro-1Hpyrrol- 1-yl)butanoic acid (0.2 mmol) in (DMF) (5 mL) at 0°C, [O-(7-azabenzotriazol-1-yl)-1,1,3,3-tetramethyluronium hexafluorophosphate] (HATU) (0.2 mmol) and 4-methylmorpholine (0.2 mmol) were added. After 10 min, [Lys(free side chain)^8^]dermorpin-resin (200 mg, 0.67 meq/gr; 0.13 meq) was added and the reaction mixture stirred at room temperature for 4 h. The solution was then filtered and the resin washed 5 times with DMF (5 ml) and 3 times with CH_2_Cl_2_ (5 ml). The protected [Lys(Mal)^8^]dermorphin-resin was treated with reagent B (trifluoroacetic acid (TFA) / H_2_0 / phenol / triisopropylsilane 88: 5: 5: 2; v/v; 10 mL / 0.2 g of resin) for 1.5 h at room temperature [23]. After filtration of the resin, the solvent was concentrated under vacuum and the residue triturated with ethyl ether. Crude peptide was purified by preparative reverse phase HPLC and lyophilized. Finally, [Cys^18^]N/OFQ-NH_2_ was reacted with [Lys(Mal)^8^]dermorphin following a procedure reported in literature and then purified by preparative HPLC to give the desired final product after lyophilisation [24].

### Cell Culture

Cells were grown in either Hams F12 (for Chinese Hamster Ovary; CHO_hMu_, CHO_hDelta_ and CHO_hKappa_ cells), DMEM/Hams F12 1:1 (for CHO_hNOP_ cells and SH-SY5Y cells) or DMEM (for HEK-293 cells). The media contained 100 μg ml^-1^ streptomycin, 2.5 μg ml^-1^ fungizone, 100 IU ml^-1^ penicillin and 10% foetal bovine serum. G418 (200 μg ml^-1^) was used to maintain CHO cells expressing classical opioid receptors. Stock media containing G418 (200 μg ml^-1^) and hygromycin B (200 μg ml^-1^) was used to maintain CHO_hNOP_ cells. HEK-293 cells permanently co-expressing the fusion proteins NOP-Rluc and Gβ1-RGFP or NOP-Rluc and β-arrestin 2-RGFP and SH-SY5Y cells co-expressing the fusion proteins Mu-Rluc and Gβ1-RGFP or Mu-Rluc and β-arrestin 2-RGFP were prepared using the pantropic retroviral expression system by Clontech as described previously [25]. Cells were used for experiments once confluent. CHO cells stably co-expressing the human recombinant NOP or Mu receptors as well as the C-terminally modified Gα_qi5_ protein were generated and used in calcium mobilisation studies as previously described [26, 27]. Cells were cultured in culture medium consisting of DMEM/Hams F12 (50/50) supplemented with 10% foetal calf serum (FCS), penicillin (100 IU/ml), streptomycin (100 mg/ml), geneticin (G418; 200 μg/ml) and hygromycin B (100 μg/ml). In all cases, cell cultures were kept at 37°C in 5% CO_2_/humidified air. The sources of cells used can be found in references [27] and [28].

### Membrane preparation

In the radioligand displacement binding assays, homogenization/wash buffer consisting of 50 mM Tris-HCl pH to 7.4 with KOH, for CHO_hMu_, CHO_hDelta_ and CHO_hKappa_ or additional 5 mM MgSO_4_ for CHO_hNOP_ was used. Homogenisation buffer (50 mM Tris and 0.2 mM EGTA pH 7.4 with NaOH) was used in GTPγ[^35^S] assays. Membranes were centrifuged at 20374 g for 10 min at 4°C. This process was repeated at least three times. The resulting pellet was resuspended in an appropriate amount of the necessary buffer and the protein concentration was determined by Lowry assay [29].

### Displacement Binding Assay

Membrane protein (40 μg) was incubated in 0.5ml of 50 mM Tris, 0.5% BSA and ~0.8nM [^3^H]-DPN (for CHO_hMu_, CHO_hDelta_ and CHO_hKappa_) or ~0.8nM [^3^H]-UFP-101 (for CHO_NOP_ cells), as well as varying concentrations (10 μM-1pM) of the reference ligand DeNo. Non-specific binding was determined in the presence of 10 μM naloxone for CHO_hMu_, CHO_hDelta_ and CHO_hKappa_ or 1μM of N/OFQ for CHO_NOP_ cells. Samples were incubated for 1 hr at room temperature and reactions were terminated by vacuum filtration, onto PEI-soaked Whatman GF/B filters, using a Brandel harvester. The concentration of displacing ligand producing 50% displacement was corrected for the competing mass of radioligand to yield pK_i_, a measure of its affinity [3].

### Calcium mobilisation assay

When confluence was reached (3–4 days), cells were seeded at a density of 50,000 cells/well into 96-well black, clear bottom plates. After 24 h incubation, the cells were loaded with medium supplemented with 2.5 mM probenecid, 3 μM of the calcium sensitive fluorescent dye Fluo-4 AM and 0.01% pluronic acid, for 30 min at 37°C. Afterwards, the loading solution was aspirated and 100 μl/well of assay buffer (Hank’s balanced salt solution supplemented with 20 mM HEPES, 2.5 mM probenecid, and 500 μM Brilliant Black (Aldrich)) was added. Serial dilutions of ligands were made in Hank’s balanced salt solution / HEPES (20 mM) buffer (containing 0.02% BSA fraction V). After placing both plates (cell culture and compound plate) into the FlexStation II (Molecular Device, Union City, CA 94587, US), fluorescence changes were measured at 37°C. Online additions were carried out in a volume of 50μl/well. Maximum change in fluorescence, expressed in percent of baseline fluorescence, was used to determine agonist response [27].

### GTPγ[^35^S] binding Assay

Membrane protein (40 μg) was incubated in 0.5 ml volume of 50 mM Tris, 0.2 mM EGTA, 1 mM MgCl_2,_ 100 mM NaCl, 0.1% BSA, 0.15 mM bacitracin; pH 7.4, GDP (33 μM), and ∼150 pM GTPγ[^35^S]. Varying concentrations of reference ligands (dermorphin, N/OFQ, Dynorphin-A and Leu-enkephalin) or DeNo (1 pM—10μM) was added prior to incubation. Non-specific binding was determined in the presence of unlabeled GTPγS (10μM). Samples were incubated at 30°C for 1 h with gentle agitation. Reactions were terminated by vacuum filtration through dry Whatman GF/B filters, using a Brandel harvester [3]

### cAMP Assay

CHO_hMu_ and CHO_hNOP_ whole cells were suspended in Krebs/HEPES buffer, containing isobutylmethylxanthine (1mM) and forskolin (1μM). For Mu, dermorphin and DeNo were included at 1μM concentrations. At NOP, N/OFQ and DeNo were included at 1μM concentrations. cAMP was extracted and assayed using a protein binding assay as described previously [30].

### Western blotting- MAPK detection

ERK1/2 and p38 MAPK activity in CHO_hMu_ and CHO_hNOP_ cells was detected by Western blotting techniques. CHO_hMu_ and CHO_hNOP_ cells were serum starved for 24 hours prior to treatment. Drugs were added for 15 minutes in Krebs Buffer [composition: 115mM NaCl, 4.7mM KCl, 2mM CaCl_2_, 1.2mM MgCl_2_, 25mM NaHCO_3_, 8mM glucose]. For Mu; dermorphin and DeNo were added at 1μM concentrations. At NOP; N/OFQ, and DeNo were added at 1μM concentrations. Signalling was terminated via Lysis Buffer [Tris-HCl (pH 7.4), 20 mM; 1% (vol/vol); Triton X-100, 10% (vol/vol); glycerol, NaCl, 137 mM; EDTA, 2 mM; β-glycerophosphate, 25 mM; sodium orthovanidate; 1 mM; phenylmethanesulfonylfluoride, 500 μM; leupeptin, 0.1 mg/ml; benzamidine, 0.2 mg/ml; pepstatin, 0.1 mg/m] followed by centrifugation (10187 g, 10 minutes, 4°C), with the supernatant removed and added to an equal volume of 2XSDS buffer [composition: 100mM Tris-HCl (pH 6.8), 2% SDS, 10% Glycerol, 0.1% Bromophenol Blue]. Samples were denatured (heated 100°C for 5 minutes) and separated by 10% SDS-PAGE; transferred onto nitrocellulose paper in a semi-dry buffer (composition: 48mM Tris Base, 39mM Glycine, 0.037% w/v SDS, 20% Methanol) and blocked using conventional western blotting techniques. To detect phosphorylated ERK1/2 and phosphorylated p38 activity, pERK1/2 antibodies (1:6000 dilution) and p38 antibodies (1:3000dilution), diluted in TBS-T solution [50 mM Tris-base, 150 mM NaCl, 0.1% Tween-20 (vol/vol), pH 7.5] with 0.01% (wt/vol) BSA, were used to probe the membrane and left overnight at 4°C. Horseradish peroxidase-conjugated secondary anti-rabbit antibodies (1hr room temperature, 1:1000 dilution in TBS-T with 5% milk) were used to visualise immune-reactive bands, followed by chemiluminescence detection using the ChemiDoc™ MP Imaging System (Bio-Rad). In order to ensure equal gel loading, membranes were stripped and reprobed for total ERK1/2 and total p38 MAPK. Membranes were incubated in Restore Plus™ (Fisher) for 15 minutes then thoroughly washed in TBS-T, following which they were blocked as previously described. The membrane was then probed using the specific antibody for ERK 1/2 (NEBL, 1:3000 dilution), or p38 (NEBL, 1:3000 dilution), overnight (4°C) followed by addition of the secondary antibody and chemiluminescence detection. Normalisation of total protein levels for each sample was then achieved by representing levels of pERK1/2 and p-p38 as a proportion of total ERK1/2 or total p38 protein. Data were analyzed using the “Origin 9” software and images analysed using Image Lab software (Bio-Rad, UK) [31].

### BRET Assay

Membrane extracts taken from HEK-293 and SH-SY5Y cells stably expressing NOP-RLuc and Mu-RLuc respectively together with Gβ_1_-RGFP were used to assess the effects of drugs on receptor/G protein interaction in concentration response curve experiments. For G-protein experiments, enriched plasma membrane aliquots from transfected cells were prepared by differential centrifugation; cells were detached with PBS/EDTA solution (1 mM, pH 7.4 NaOH) then, after 5 min 500 g centrifugation, Dounce-homogenized (30 strokes) in cold homogenization buffer (TRIS 5 mM, EGTA 1 mM, DTT 1 mM, pH 7.4 HCl) in the presence of sucrose 0.32 M. Three further centrifugations were performed at 1000 g (4°C) and the supernatants kept. Two 25,000 g (4°C) subsequent centrifugations (the second in the absence of sucrose) were performed for separating enriched membranes that, after discarding the supernatant, were kept in ultrapure water at -80°C [32]. The protein concentration in membrane preparations was determined using the QPRO-BCA kit (Cyanagen Srl, Bologna, IT) and a Beckman DU 520 spectrophotometer (Brea, CA, USA). Luminescence in membranes was recorded in 96-well untreated white opaque microplates (PerkinElmer, Waltham, MA, USA) using the Victor 2030 luminometer (PerkinElmer, Waltham, MA, USA). For the determination of receptor/G-protein interaction, membranes (3 μg of protein) prepared from cells co-expressing NOP or Mu-RLuc and Gβ1-RGFP were added to wells in Dulbecco’s PBS. For the determination of receptor/β-arrestin 2 interaction, cells co-expressing NOP-RLuc or Mu-RLuc and β-arrestin 2-RGFP were plated 24 h before the experiment in poly-D-Lysine treated plates (100,000 cells/well), while for Mu expressing cells untreated plates were used. The cells were prepared for the experiment substituting the medium with DPBS supplemented with MgCl_2_ (0.5 mM) and CaCl_2_ (0.9 mM). Coelenterazine, at a final concentration of 5 μM, was injected 15 minutes prior to reading the cell plate. Receptor/G protein interaction was measured in cell membranes to exclude the participation of other cellular processes (i.e. arrestin recruitment, internalization). Different concentrations of ligands in 20 μL of PBS—BSA 0.03% were added and incubated for an additional 5 min before reading luminescence. All experiments were performed at room temperature.

### Guinea pig ileum bioassay

With approval of Animal Subjects Review Board of the University of Ferrara and from the Italian Ministry of Health (PROT-186) ileum tissues were taken from male albino guinea pigs of 350–400 g (Pampaloni, Pisa, Italy). The animals were treated in accordance with European guidelines (86/609/ECC) and national regulations (DL 116/92). They were housed in 560 x 320 x 180 mm cages (Techinplast), three per cage, under standard conditions (22°C, 55% humidity, 12 h light/dark cycle, light on at 7:00 h) with food (complete feed for guinea pig, Mucedola, Milano, Italy) and water *ad libitum*. The day of the experiment the animals were killed with an isofluorane overdose. Bioassay experiments were performed as previously described [33]. The tissues were suspended in 5 ml organ baths containing Krebs solution (composition in mM: NaCl 118.5, KCl 4.7, MgSO_4_ 1.2, KH_2_PO_4_ 1.2, NaHCO_3_ 25, CaCl_2_ 1.8, glucose 10), hexamethonium bromide 22 μM, benadril 0.34 μM and oxygenated with 95% O_2_ and 5% CO_2_. The temperature was set at 37°C. At resting tension, 1 g was applied to the tissues. Tissues were stimulated through two platinum electrodes with a supramaximal rectangular pulse of 1 ms duration, 0.05 Hz frequency, 80V amplitude. Electrically evoked contractions were measured isotonically by means of Basile strain gauge transducers (Basile 7006; srl Ugo Basile, Varese, Italy) and recorded with a computer–based acquisition system (Power Lab 8, ADInstruments, USA). After an equilibration period of about 60 min, the contractions induced by electrical field stimulation were stable. At this time, cumulative concentration response curves to agonists were constructed (0.5 log unit steps). Antagonists were injected into the baths 15 minutes before constructing agonist concentration response curves.

### In Vivo Studies

For all the experiments described below, male Sprague-Dawley rats (Harlan, Varese, Italy), weighing approximately 280-300g at the beginning of the experimental procedure, were used. Animals were housed in CeSAL (Centro Stabulazione Animali da Laboratorio, University of Florence) and used at least one week after their arrival. One rat was housed per cage (size 26x41 cm); animals were fed with standard laboratory diet and tap water *ad libitum*, and kept at 23±1°C with a 12 h light/dark cycle, light at 7 a.m. All animal manipulations were carried out according to the European Community guidelines for animal care (DL 116/92, application of the European Communities Council Directive of 24 November 1986 (86/609/EEC). The ethical policy of the University of Florence complies with the Guide for the Care and Use of Laboratory Animals of the US National Institutes of Health (NIH Publication No. 85–23, revised 1996; University of Florence assurance number: A5278-01). Formal approval to conduct the experiments described was obtained from the Animal Subjects Review Board of the University of Florence and from the Italian Ministry of Health (N°54/2014-B). Experiments involving animals have been reported according to ARRIVE guidelines [34]. All efforts were made to minimize animal suffering and to reduce the number of animals used. All animals were monitored daily using a scoring system (based on: Appearance, Food and Water Intake, Clinical Signs, Natural Behaviour and Provoked Behaviour [35]. Maximum score is 20 and animals reaching 10 are euthanized. In the experiments reported here no animals reached this score and none died before the end of the experiment) and at the end of the experiment were euthanized by CO_2_ overdose.

### Intrathecal catheterization

Rats were anaesthetised with 2% isoflurane and an intrathecal catheter was surgically implanted according to the method of Yaksh & Rudy (1976) [36]. Rats were shaved on the back of the neck and placed in the stereotaxic frame with the head securely held between ear bars. The skin over the nape of the neck was cleaned with ethyl alcohol and incised for 1 cm. The muscle on either side of the external occipital crest was detached and retracted to expose about 3–4 mm^2^ of the atlanto-occipital membrane. The membrane was incised by a needle, which led to the escape of cerebrospinal fluid. The caudal edge of the cut was lifted and about 7.0 cm of 28G polyurethane catheter (0.36 mm outer diameter; 0.18 mm inner diameter; Alzet, USA) was gently inserted into the intrathecal space in the midline, dorsal to the spinal cord until the lumbar enlargement. The exit end of the catheter was connected to 4.0 cm polyurethane tube (0.84 mm outer diameter; 0.36 mm inner diameter) and was taken out through the skin, flushed with saline solution, sealed and securely fixed on the back of the head with a silk wire. Animals were placed in individual cages until recovery. All animals used during behavioural tests did not show surgery induced motor impairment as evaluated by Rota rod test. Animals presenting any kind of motor disability were excluded from the behavioural measurements. Behavioral measurements were performed on 5 rats for each treatment.

### Intrathecal drug treatments

Dermorphin and DeNo were dissolved in sterile saline solution. Acute measures were performed after the intrathecal (i.t.) infusion of 0.1–1 nmol dermorphin and DeNo. All compounds were administered in a final volume of 10 μl. Behavioral tests were carried out after 15, 30, 45, 60, 90, 120 and 180 min.

### Paw Pressure test

Nociceptive threshold was determined with an analgesimeter (Ugo Basile, Varese, Italy), according to the method described by Leighton et al. (1988) [37]. Briefly, a constantly increasing pressure was applied to a small area of the dorsal surface of the paw using a blunt conical probe by a mechanical device. Mechanical pressure was increased until vocalization or a withdrawal reflex occurred while rats were lightly restrained. Vocalization or withdrawal reflex thresholds were expressed in grams. Rats scoring below 40 g or over 75 g during the test before drug administration were rejected. For analgesia measures, mechanical pressure application was stopped at 150 g. All experiments were performed by a researcher blind to drug treatment.

### Rota-rod test

Rota-rod apparatus (Ugo Basile, Varese, Italy) consisted of a base platform and a rotating rod with a diameter of 6 cm and a non-slippery surface. The rod was placed at a height of 25 cm from the base. The rod, 36 cm in length, was divided into 4 equal sections by 5 disks. Thus, up to 4 rats were tested simultaneously on the apparatus, with a rod-rotating speed of 10 rpm. The integrity of motor coordination was assessed on the basis of the number of falls from the rod in 60s measured 15, 30, 45, 60, 90, 120 and 180 min after treatments.

### Data analysis

Data are expressed as mean±SEM or with confidence intervals as appropriate. For more than 2 groups, data are analysed using ANOVA with post-hoc testing using Dunnett’s or Bonferroni’s test as appropriate. Where there are only 2 groups paired or unpaired t-tests were used. *P* values of less than 0.05 were considered significant. All curve fitting was accomplished using Graphpad-Prism (V6). The concentration of drug producing 50% of the maximum response (pEC_50_) and the maximum response (E_max_) are quoted. In gpI experiments the antagonist potency (pK_b_) is calculated from the rightward shift of the agonist concentration response curve by a fixed antagonist concentration.

## Results

### Displacement Binding assay

**Fig 2:**
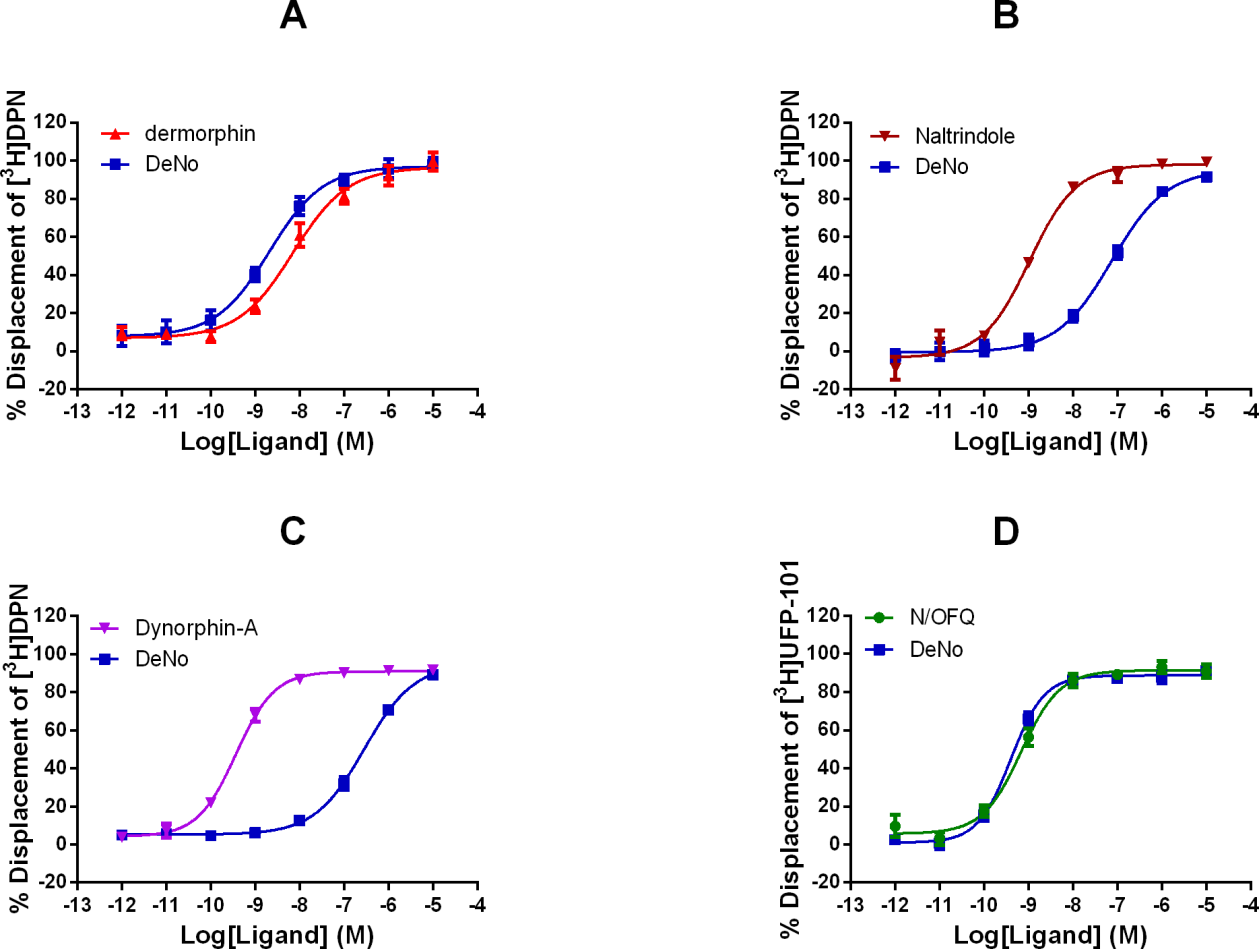
Displacement Binding. Displacement of tritiated Diprenorphine ([^3^H]-DPN) by dermorphin, DeNo, and reference ligands at (A) CHO_hMu,_ (B) CHO_hDelta_, (C) CHO_hKappa_ and the displacement of tritiated UFP-101 ([^3^H]-UFP-101) by DeNo and N/OFQ at (D) CHO_NOP_ cell membranes. Data are means (SEM) of n≥5 experiments for all cell lines. Reference ligands: dermorphin; naltrindole; dynorphin-A; N/OFQ, (nociception/orphanin FQ).

In displacement binding studies at CHO_hMu_, dermorphin and DeNo displaced the binding of [^3^H]-DPN in a concentration dependent and saturable manner (Fig 2). DeNo (pK_i_ 9.55) demonstrated a significant increase in affinity at Mu, when compared to the parent compound dermorphin (8.69). At CHO_hNOP_, DeNo displaced [^3^H]-UFP-101 in a concentration dependent and saturable manner (Fig 2). DeNo (10.22) displayed a similar pK_i_ value, for NOP, to its parent compounds N/OFQ (10.69) (Fig 2, Table 1). At CHO_hDelta_, DeNo (8.12) demonstrated an increase in affinity compared to its parent compounds. Dermorphin displayed an affinity of 7.17, while N/OFQ failed to displace [^3^H]-DPN at the Delta receptor. Furthermore, DeNo (7.34) showed affinity for the Kappa receptor, whereas the parent compounds (dermorphin and N/OFQ) failed to displace [^3^H]-DPN at this receptor (Fig 2, Table 1).

**Table 1:** Displacement Binding Profiles.

pK_i_				
	CHO_hMu_	CHO_hNOP_	CHO_hDelta_	CHO_hKappa_
**Reference ligand**	-	-	10.02 _(±0.26)_	10.16 _(±0.02)_
**dermorphin**	8.69 _(±0.10)_	<5	7.17 _(±0.11)_	<5
**N/OFQ**	<5	10.69 _(±0.10)_	<5	<5
**DeNo**	9.55 _(±0.10)_*	10.22 _(±0.09)_	8.12 _(±0.11)_*	7.34_(±0.13)_*

pK_i_ values for both the reference ligands, dermorphin, N/OFQ and DeNo. Data are displayed as mean (±SEM) of n = 5 experiments. Statistical significance (*) demonstrates p<0.05, using one-way ANOVA with Dunnett’s correction, when compared to the reference ligands dermophin (Mu), N/OFQ (NOP), Naltrindole (Delta) and Dynorphin-A (Kappa).

### Calcium mobilisation assay

**Fig 3:**
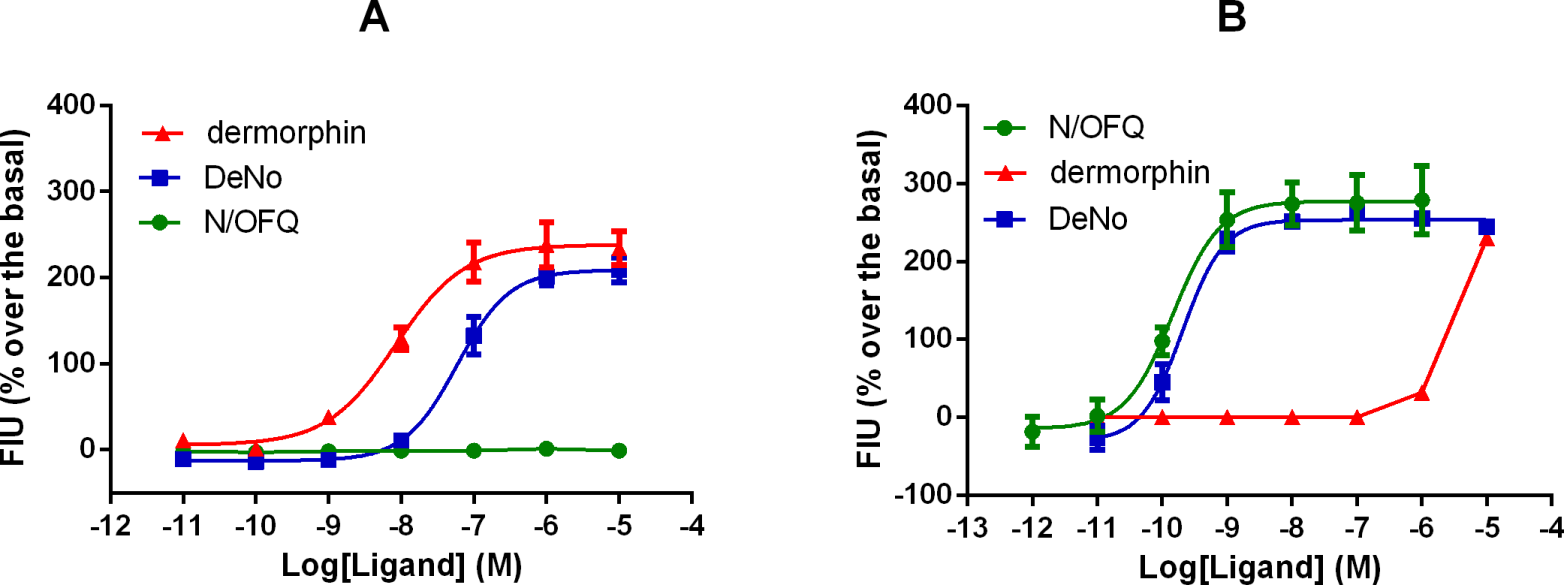
Calcium Mobilisation. Concentration-response curves to N/OFQ, dermorphin and DeNo in calcium mobilisation assay using CHO_hMu_ cells (A) and CHO_NOP_ cells (B). Data is represented as percentage increase over basal fluorescence intensity units (FIU). Data are the mean ± SEM of at least three separate experiments.

In CHO cells stably expressing the Gα_qi5_ chimeric protein and the human Mu receptor, dermorphin produced a concentration-dependent stimulation of calcium mobilisation with high potency (pEC_50_ 8.07) and maximal effects (234% over basal). DeNo showed similar maximal effect (209% over basal) but approximately 10-fold lower potency (pEC_50_ 7.17) (Fig 3). In CHO_hNOP_ cells stably expressing the Gα_qi5_ chimeric protein, N/OFQ evoked a concentration dependent stimulation of calcium release with high potency (pEC_50_ 9.85) and maximal effect (279% over basal). DeNo showed similar potency (pEC_50_ 9.69) and maximal effect (244% over basal) to N/OFQ (Fig 3 and Table 2).

**Table 2:** Calcium Mobilisation.

	NOP		Mu	
	pEC_50 (CL95%)_	E_max ± SEM_	pEC_50 (CL95%)_	E_max ± SEM_
**N/OFQ**	9.85(_9.37–10.33)_	279 ± 44%	Inactive	
**dermorphin**	crc incomplete		8.07_(7.85–8.29)_	234 ± 14%
**DeNo**	9.69_(9.35–10.03)_	244 ± 6%	7.17_(6.91–7.43)_	209 ± 15%

Effects of N/OFQ, dermorphin and DeNo in calcium mobilisation experiments performed in CHO cells stably expressing the human NOP or Mu receptor and the G_αqi5_ protein. Data are expressed as the mean (±SEM) of at least 3 experiments performed in duplicate. For potency values 95% confidence limits were indicated. Data have been analysed with one way ANOVA followed by the Dunnett’s test for multiple comparisons; p values less than 0.05 were considered to be significant.

### GTPγ[^35^S] Assay

**Fig 4:**
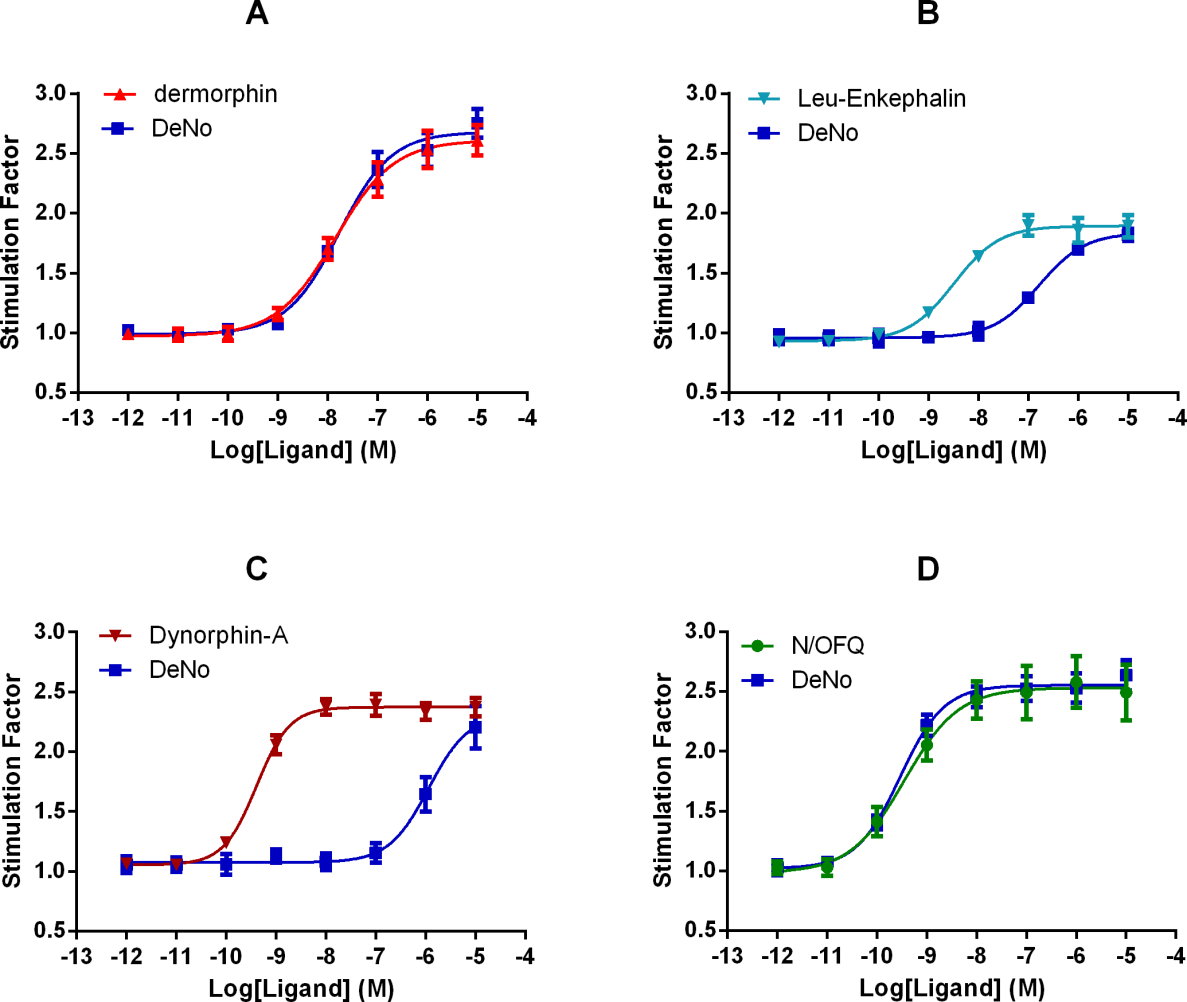
GTPγ[^35^S] Response Curves. Ligand stimulated GTPγ[^35^S] binding by DeNo and reference ligands are shown at A) CHO_hMu,_ (B) CHO_hDelta_, (C) CHO_hKappa_ and (D) CHO_NOP_ cell membranes. Graphs are represented as a stimulation factor, which is the fold change in activity when compared to basal. Data are mean (±SEM) for n = 5 experiments.

Dermorphin and DeNo stimulated the binding of GTPγ[^35^S] in a concentration dependent and saturable manner at the Mu receptor (Fig 4). DeNo (E_max_ 2.68) demonstrated a similar maximal response to that of dermorphin (2.63). The pEC_50_ values for DeNo (7.77) showed no significant difference to that of the parent compound, dermorphin (7.83) (Fig 4, Table 3). At CHO_hNOP_, N/OFQ and DeNo stimulated the binding of GTPγ[^35^S] in a concentration dependent and saturable manner (Fig 4). DeNo produced a maximal response (E_max_ 2.49) similar to that of its parent compound, N/OFQ (2.57). The pEC_50_ value of 9.50 achieved by DeNo was similar to that of N/OFQ (9.05). (Table 3). Leu-enkephalin and DeNo stimulated the binding of GTPγ[^35^S] in a concentration dependent and saturable manner in membranes expressing Delta receptors (Fig 4). DeNo (E_max_ 1.84) produced a maximal response similar to that of the endogenous Delta peptide, Leu-enkephalin (1.90). However, the pEC_50_ value for DeNo (6.78) was significantly lower than that of Leu-enkephalin (8.50) (Table 3). At CHO_hKappa_, dynorphin-A and DeNo stimulated the binding of GTPγ[^35^S] in a concentration dependent and saturable manner (Fig 4). DeNo (E_max_ 2.36) produced a maximal response similar to that of dynorphin-A (2.33). The pEC_50_ value of DeNo (5.91) was significantly lower than that of dynorphin-A (9.36) (Table 3).

**Table 3:** GTPγS Functional Activity.

	CHO_hMu_			CHO_hNOP_			CHO_hDelta_			CHO_hKappa_		
	pEC_50_	E_max_	α	pEC_50_	E_max_	α	pEC_50_	E_max_	α	pEC_50_	E_max_	α
**Control**	7.83_(7.45–8.21)_	2.61_(±0.14)_	1	9.05_(8.77–9.34)_	2.57_(±0.17)_	1	8.5_(8.19–8.81)_	1.9_(±0.10)_	1	9.34(9.22–9.49)	2.3(±0.09)	1
**DeNo**	7.77_(7.54–7.80)_	2.68_(±0.12)_	1.03	9.5_(9.33–9.68)_	2.49_(±0.25)_	0.96	6.78_(6.59–6.97)_*	1.84_(±0.03)_	0.97	5.92(5.78–6.02)*	2.23(±0.14)	0.97

Agonist activity of DeNo at CHO_hMu_, CHO_hNOP,_ CHO_hDelta_ and CHO_hKappa_. Antagonist affinity was determined against N/OFQ (NOP). Relative intrinsic activity (α) was determined by removal of basal activity and as a ratio of reference ligands (full agonist) E_max_. All experiments are represented as the mean (±SEM) of n≥5 experiments. For potency values 95% confidence limits were indicated.

* p<0.05 t-test against the reference ligand [Dermophin (Mu), N/OFQ (NOP), Naltrindole (Delta) and Dynorphin-A (Kappa)].

### Cyclic AMP assay

To further assess functional activity of DeNo, inhibition of cyclic AMP (cAMP) formation was assessed. Since DeNo demonstrated a higher affinity and potency at Mu and NOP, assays were performed using CHO_hMu_ and CHO_hNOP_ cells only. Addition of forskolin in CHO_hMu_ cells lead to a 24.3 (±1.79) fold increase in cAMP production, when compared to basal activity (Fig 3). Co-incubation of 1μM dermorphin, or 1μM DeNo reverses the effects of forskolin, returning cAMP levels to basal. In CHO_hNOP_ cells, forskolin stimulation leads to a 21.23 (±3.86) fold increase in cAMP production, when compared to basal activity. The addition of 1μM N/OFQ reverses forskolin stimulated cAMP production. The addition of 1μM DeNo has a similar effect, returning cAMP levels to basal.

### Detection of MAPK activity through Western blot Densitometry

**Fig 5:**
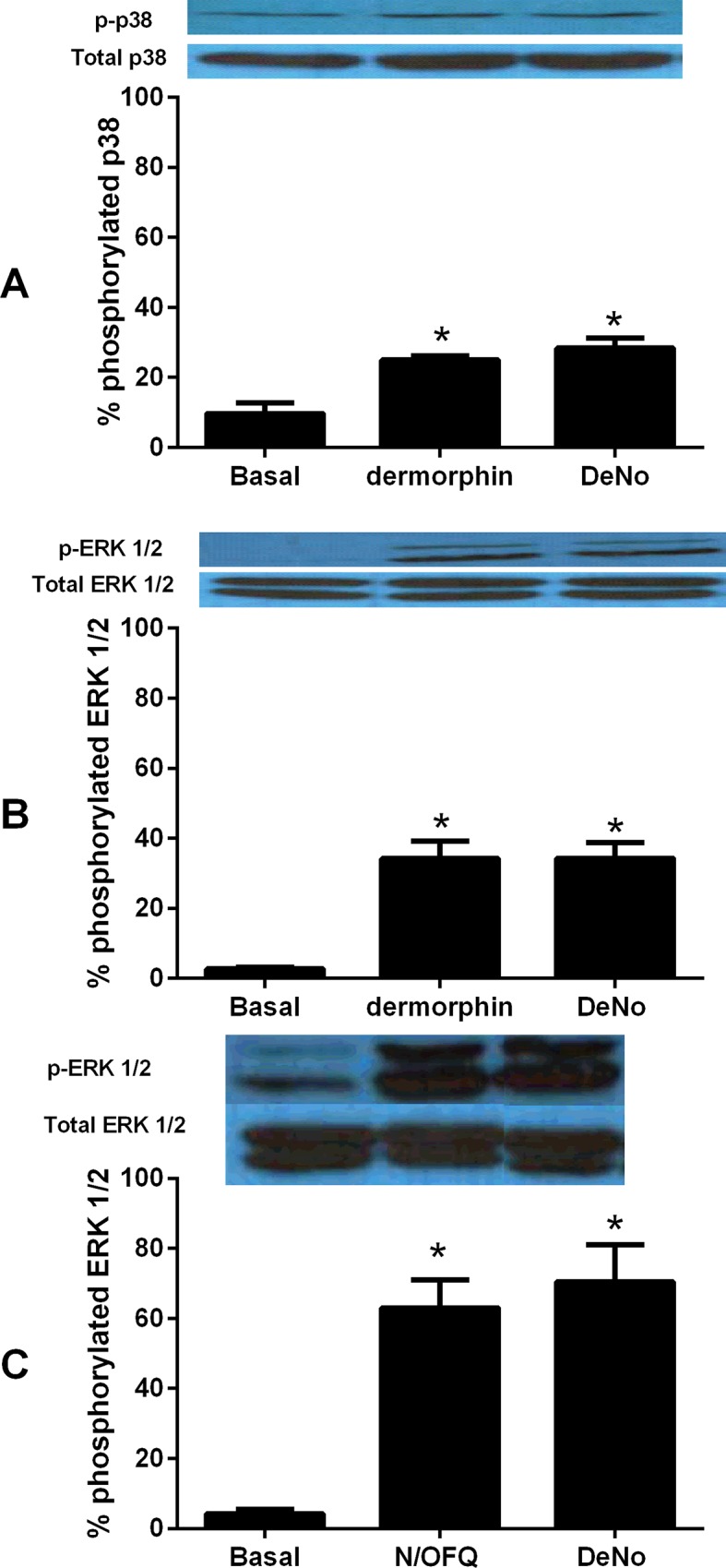
The Activation of ERK1/2 and p38. A) The activity of phosphorylated p38 compared to total p38 at CHO_hMu_ caused by dermorphin (1μM) and DeNo (1μM). B) The activity of phosphorylated ERK1/2 compared to total ERK1/2 at CHO_hMu_ caused by dermorphin (1μM) and DeNo (1μM). C) The activity of phosphorylated ERK1/2 at CHO_hNOP_ caused by N/OFQ (1μM) and DeNo (1μM). All data are after a 15 minute incubation period. Data are mean (±SEM) for n = 5. *p<0.05; according to ANOVA followed by Dunnett’s test for multiple comparison. Representative blots are shown above mean data.

In CHO_hMu_ cells, basal activity of phosphorylated p38 (p-p38) was 9.67±1.39% of total. Following stimulation by 1μM dermorphin, this activity rose to 25.02±1.18%, this was statistically significant. Administration of DeNo (28.41±1.27%) led to a similar increase in p-p38 activity. In phosphorylated ERK1/2 (p-ERK1/2) assays, basal activity was measured at 2.75 ± 0.83% of total. The addition of 1 μM dermorphin (34.23 ± 4.97%) or DeNo (34.30 ± 4.5%) produced a statistically significant increase over basal activity (Fig 5). In CHO_hNOP_ cells, phosphorylation of p38 was not detected. In studies assessing the activation of p-ERK1/2, basal activity was measured at 4.24 ±1.26% of total. The addition of 1μM N/OFQ (63.08 ±7.97%) or DeNo (70.46 ±10.63%) produced a statistically significant increase over basal activity (Fig 5).

### BRET Assays

**Fig 6:**
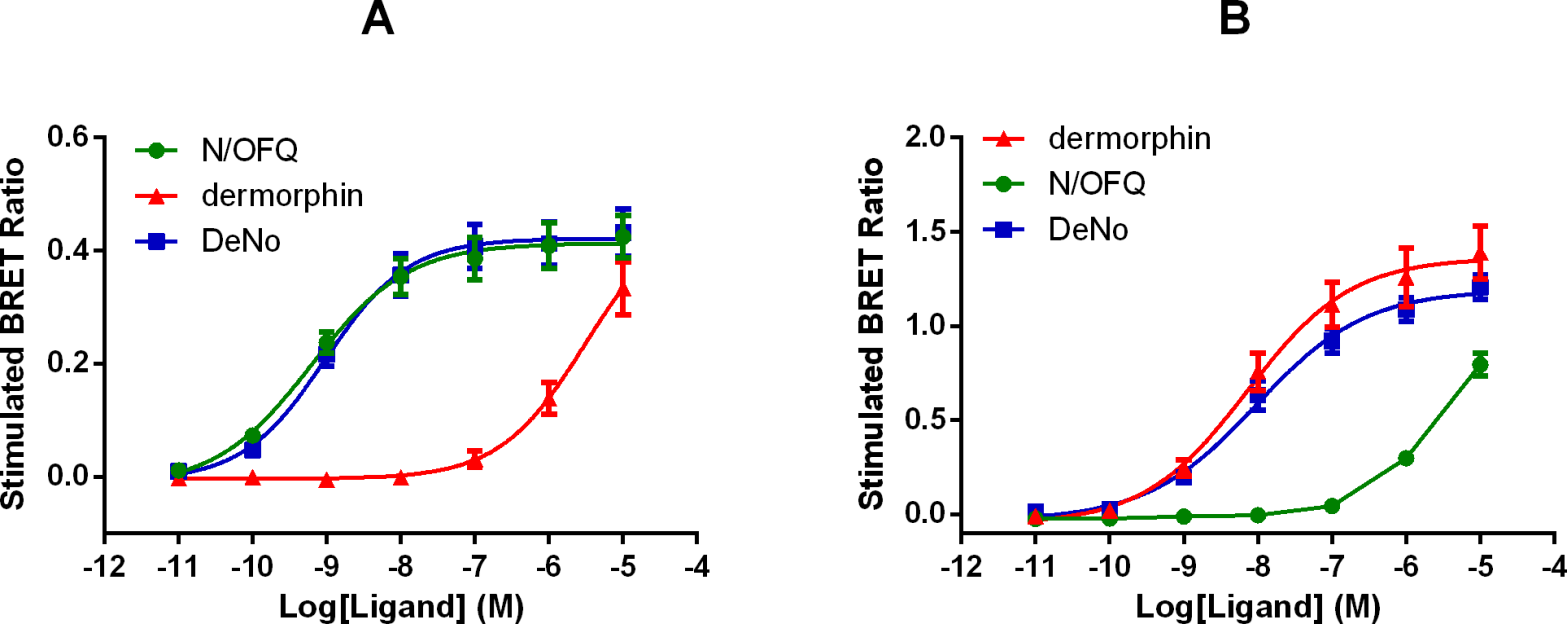
G-protein Recruitment Measured Using BRET. Membrane extracts taken from HEK-293 and SH-SY5Y cells stably expressing NOP-RLuc and Mu-RLuc respectively together with Gβ_1_-RGFP were used. Concentration response curves to N/OFQ, dermorphin and DeNo for receptor/G protein interaction in membranes of cells expressing the NOP (A) and Mu (B) receptors. Data are the mean ± SEM of at least 3 experiments performed in duplicate.

In HEK-293 membranes, N/OFQ promoted NOP-RLuc/G-protein-RGFP interaction in a concentration-dependent manner with high potency (pEC_50_ 9.22) and a maximal effect corresponding to 0.42±0.04 stimulated BRET ratio. Dermorphin was very weak only increasing BRET ratio at micromolar concentrations (Fig 6, Table 4). In SH-SY5Y membranes, dermorphin produced a concentration dependent stimulation of G-protein interaction, also with high potency (pEC_50_ 8.13). In this cell type, the maximal effect was larger at 1.39±0.14 stimulated BRET ratio. N/OFQ was very weak increasing BRET ratio only at micromolar concentrations. Under the same experimental conditions, DeNo was tested in both cells lines. In HEK-293 membranes, DeNo mimicked the stimulatory effect of N/OFQ with similar potency (9.02) and maximal effect (α 1.01) (Fig 6). In SH-SY5Y membranes, DeNo concentration dependently increased BRET ratio with similar potency and maximal effect (pEC_50_ 8.03 and α 0.98) to dermorphin (Fig 6, Table 4). HEK-293 and SH-SY5Y cells stably expressing the human NOP or Mu (NOP/Mu-RLuc) receptors and β-arrestin 2-RGFP were used to evaluate NOP and Mu interaction with β-arrestin 2.

**Table 4:** Assessment of G-protein and Receptor Interactions Using BRET.

	Gβ1-protein			
	NOP		Mu	
	pEC_50(CL95%)_	α _± SEM_	pEC_50 (CL95%)_	α _± SEM_
**N/OFQ**	9.22_(9.14–9.30)_	1	crc incomplete	
**dermorphin**	crc incomplete		8.13_(7.87–8.39)_	1
**DeNo**	9.02_(8.87–9.17)_	1.01 _± 0.01_	8.01_(7.39–8.63)_	0.98 _± 0.03_

Effects of compounds tested on NOP/Gβ1- HEK-293 and Mu/Gβ1- SH-SY5Y protein interactions. Data are expressed as the mean (±SEM) of at least 3 experiments performed in duplicate. For potency values 95% confidence limits were indicated. Maximal effects elicited by the ligands were expressed as intrinsic activity (α) related to the standard control.

**Fig 7:**
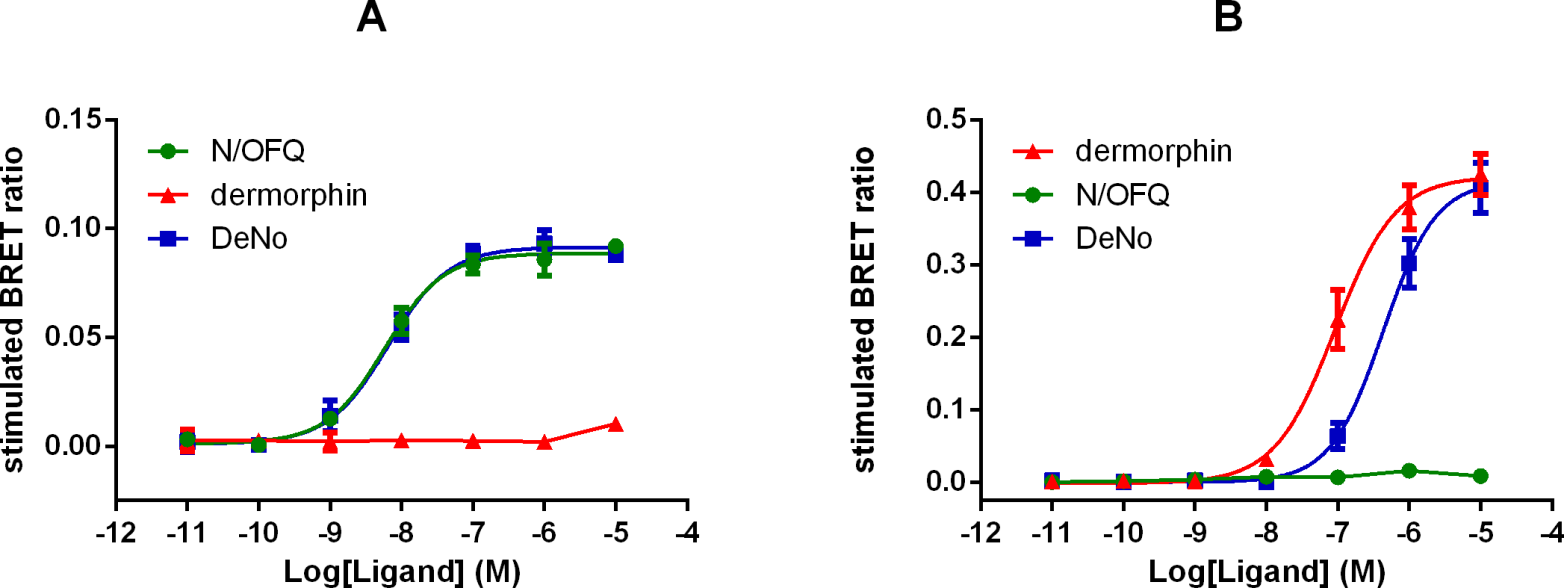
β-arrestin 2 recruitment measured using BRET. Membrane extracts taken from HEK-293 and SH-SY5Y cells stably expressing NOP-RLuc and Mu-RLuc respectively together with β-arrestin 2-RGFP were used. Concentration response curves to N/OFQ, dermorphin and DeNo for receptor/β-arrestin 2 interaction in cells expressing the NOP (panel A) and Mu (panel B) receptors. Data are the mean ± SEM of at least 4 experiments performed in duplicate.

In HEK-293 cells, N/OFQ promoted NOP/β-arrestin 2 interaction with high potency (pEC_50_ 8.21) and maximal effect corresponding to 0.092±0.003 stimulated BRET ratio. Dermorphin was completely inactive (Fig 7, panel A). In SH-SY5Y cells, dermorphin stimulated Mu β-arrestin 2 interaction with a potency (pEC_50_) of 7.00. Maximal effect was 0.43 stimulated BRET ratio (again in this cell line simulated ratio was larger). N/OFQ was completely inactive (Fig 7). Under the same experimental conditions in HEK-293 cells DeNo mimicked the stimulatory effect of N/OFQ (pEC_50_ 8.19 and α 1.03). In SH-SY5Y cells, DeNo promoted Mu/β-arrestin 2 interaction mimicking the stimulatory effect of dermorphin (α 0.98) but with 4 fold lower potency (pEC_50_ 6.36) (Fig 7, Table 5).

**Table 5:** β-arrestin Recruitment.

	β-arrestin 2			
	NOP		Mu	
	pEC_50 (CL95%)_	α _± SEM_	pEC_50 (CL95%)_	α _± SEM_
**N/OFQ**	8.21_(7.97–8.45)_	1	inactive	
**dermorphin**	inactive	inactive	7_(6.59–7.42)_	1
**DeNo**	8.19_(7.93–8.46)_	1.03 _± 0.02_	6.36_(6.08–6.63)_	0.98 _± 0.04_

Effects of compounds tested on NOP/β-arrestin 2—HEK-293 and Mu/ β-arrestin 2 SH-SY5Y protein interactions. Data are expressed as the mean (±SEM) of at least 4 experiments performed in duplicate. For potency values 95% confidence limits were indicated. Maximal effects elicited by the ligands were expressed as intrinsic activity (α) related to the standard control.

### Guinea pig ileum bioassay

**Fig 8:**
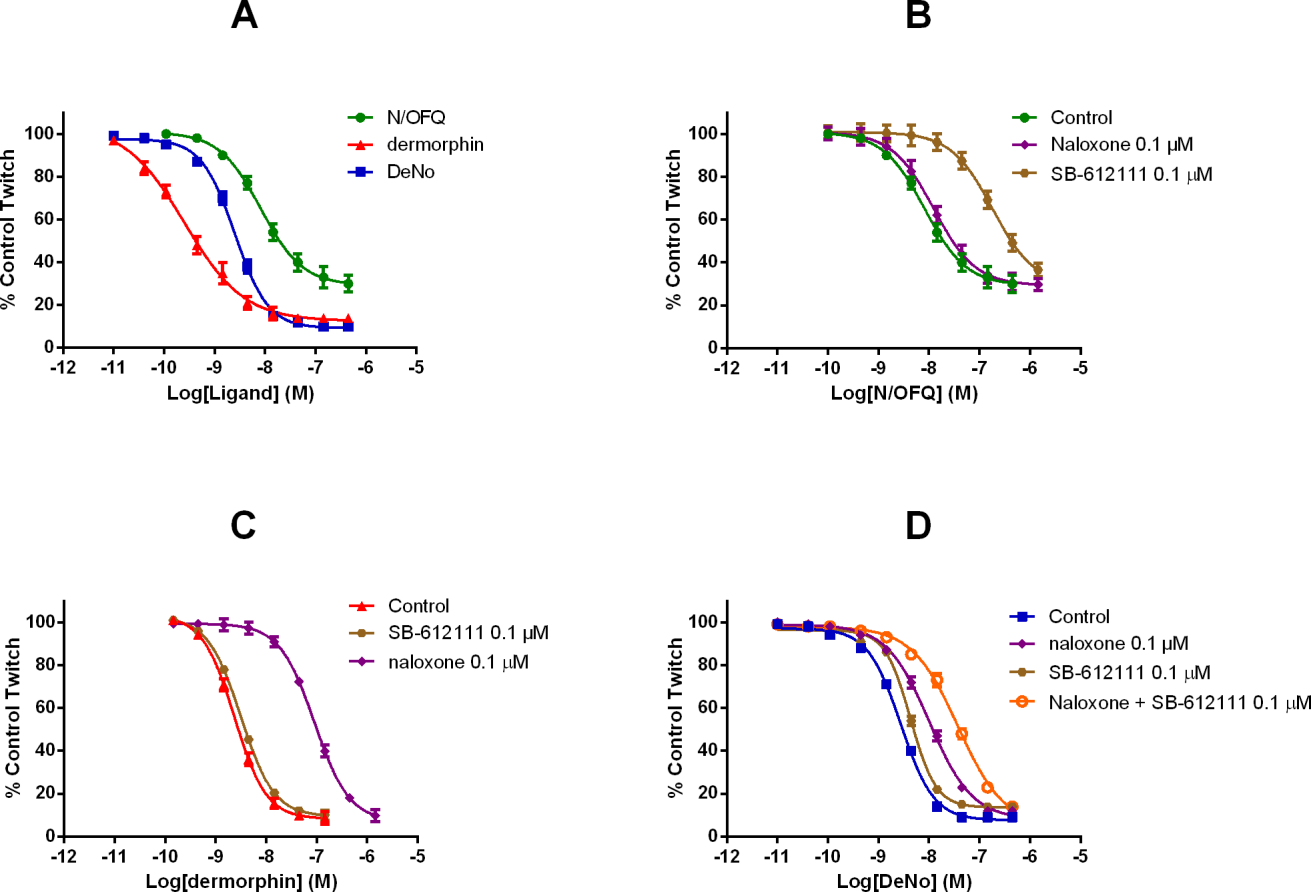
Guinea Pig Ileum Contractile Response. (A) Concentration-response curves to N/OFQ, dermorphin and DeNo in the electrically stimulated guinea-pig ileum. Effects of naloxone, SB-612111 and a cocktail of both on concentration-response curves to N/OFQ (B), dermorphin (C) and DeNo (D) in electrically stimulated guinea-pig ileum. Data are presented as a % of control twitch and the mean ± SEM for at least n = 3.

DeNo was assessed in the electrically stimulated guinea pig ileum. In this preparation, concentration response curves to DeNo were assessed in comparison with N/OFQ and dermorphin (Fig 8). N/OFQ inhibits contractions induced by electrical field stimulation in a concentration dependent manner, with a pEC_50_ of 8.03 (7.84–8.23) and maximal effect of 71±4%. Dermorphin mimicked the effects of N/OFQ with higher potency and maximal effects (pEC_50_ 9.55 (9.33–9.77) E_max_ 86 ± 2%). The new compound, DeNo, inhibited electrically induced twitch, with a potency of 8.63 and maximal effects similar to those of dermorphin (E_max_ 90 ± 1%).

In order to determine the site of action of DeNo in the guinea pig ileum, antagonist assays were undertaken. The standard non-selective opioid antagonist naloxone (100 nM) does not affect the inhibitory action of N/OFQ, while 100 nM SB-612111 was able to shift to the right the concentration response curve to N/OFQ with a pK_b_ of 8.36. In contrast, the effects of dermorphin were sensitive to naloxone (pK_b_ 8.57) but not to SB-612111 (Fig 8). Finally, the effects of DeNo were challenged with naloxone, SB-612111, and the cocktail of the two antagonists. Naloxone antagonized the inhibitory effect of DeNo producing a rightward shift of the concentration response curve and no modification of maximal effects. A pK_b_ value of 7.54 was derived from these experiments. SB-612111 was also able to counteract DeNo effects by producing a displacement to the right of the concentration response curve; a pK_b_ value of 7.07 was derived from these experiments. When the two antagonists were assayed together they displayed a clear additive effect.

### Paw pressure Test

**Fig 9:**
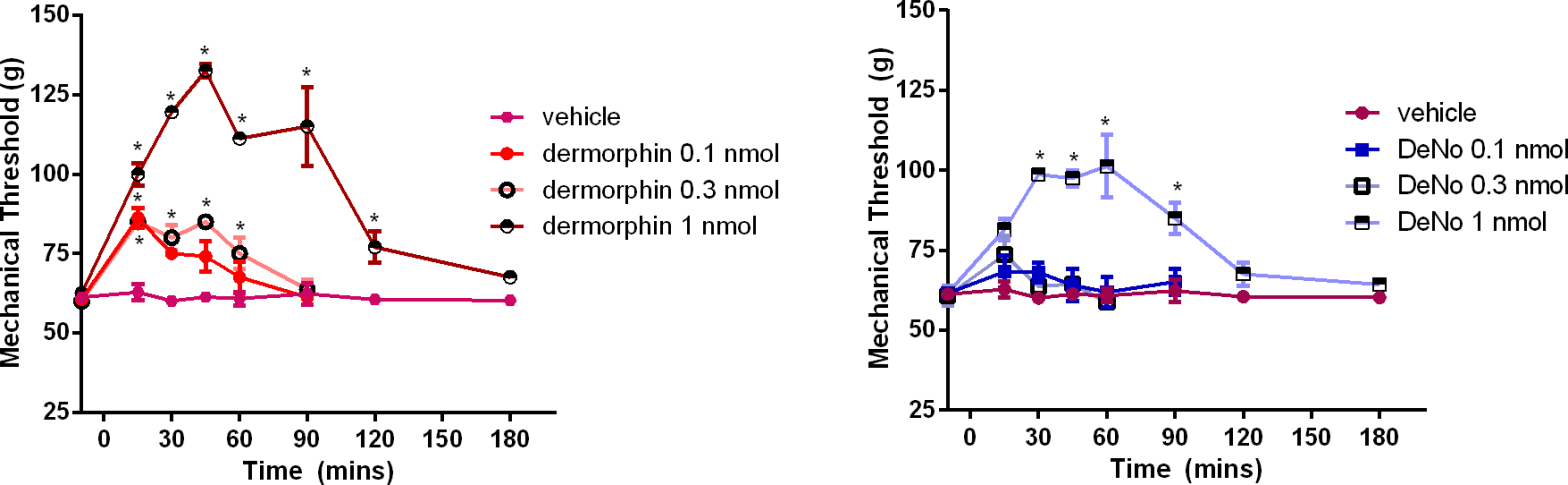
*In Vivo* activity of DeNo. Dose response curve to dermorphin (panel A) and DeNo (panel B) in rats subjected to the paw pressure test. Data (as mechanical threshold measured in g) are the mean ± SEM of 5 rats in each group. *p<0.05, according to one way ANOVA followed by the Dunnett’s test for multiple comparisons. An increase is indicative of an antinociceptive (or analgesic) response.

Time-courses were produced for both dermorphin and DeNo given intrathecally (i.t.) in rats subjected to the plantar test. Dermorphin produced significant antinociceptive effects (Fig 9A). Similar effects were measured in response to i.t. DeNo which was less potent producing statistically significant effects only at the top dose of 1 nmol (Fig 9B). However it should be ubderscored that in the same range of doses both compounds impaired animal performance in the rotarod test (data not shown).

## Discussion

In this study we have characterised a novel peptide bivalent Mu-NOP ligand, DeNo; created by combining dermorphin and N/OFQ. DeNo bound to Mu and NOP receptors and was ~5fold NOP selective. There was between 1 and 2 logs selectivity over Delta and Kappa receptors. At conventional upstream (GTPγ[^35^S]) and downstream (cAMP) assays and in Ca^2+^ mobilisation experiments using chimeric G-protein constructs DeNo was a full agonist at both Mu and NOP. In BRET assays to assess receptor G-protein interaction and arrestin recruitment, DeNo also behaved as a full agonist. Moreover, DeNo activated ERK1/2 as a full agonist at Mu and NOP; there was no activation of p38 at NOP. In a more intact preparation DeNo inhibited contraction of the electrically stimulated gpI via simultaneous activation of NOP and Opioid receptors and was antinociceptive via the i.t. route in rats.

At the receptor, the most striking difference was a log increase in DeNo binding affinity at Mu compared to dermorphin and this may result from the structure of the linker between the two ‘pharmacophores’. Comparing CHO data and taking into account different assays/buffer systems there was a marked difference in pK_i_ / pEC_50_ for Mu falling 60 fold at GTPγ[^35^S] (upstream) and 331 fold at the chimeric G-protein (downstream). In contrast at NOP there was a 7 and 3 fold difference in affinity compared to potency. As the point of interrogation (assay) moved further down the signal transduction cascade the difference in potency at Mu and NOP increased. For example in GTPγ[^35^S] assay DeNo was 38 fold more potent at NOP and 331 fold more potent in the chimeric G-protein assay. In all assays DeNo was a full agonist. If there was amplification or a coupling reserve then the potency values should shift leftwards rather than rightwards. This rightward shift was more marked at Mu than NOP and could be explained by buffer composition and end point. It is well documented that the chimeric G-protein assay suffers from hemi-equilibrium problems and we have discussed this in detail previously [38].

Using BRET based assays, we have been able to assess receptor/G protein interaction and arrestin recruitment. These end points were examined in HEK cells for NOP and SH-SY5Y cells for Mu; this is a minor drawback in comparison with the other assays reported but because we have used dermorphin and N/OFQ then we can cross compare. In all assays, the parent compounds and DeNo behaved in essentially the same manner (potency) and as full agonists. A possible confounder is that SH-SY5Y cells have been shown to express both the Delta receptor and, more relevantly, the NOP receptor; at very low expression levels [39]. Previous work with Mu/NOP heterodimers has demonstrated that activation of NOP often leads to a reduction in the potency of Mu agonists throughout the cell signalling cascade [15]. It is possible in this cell line that DeNo is engaging both Mu and NOP. It has been known for some time that opioid receptors couple to MAPK with some variation in coupling [40, 41]. In CHO cells expressing recombinant Mu and NOP there were marked differences. ERK1/2 was ubiquitously activated but p38 was only activated by Mu. ERK1/2 is typically involved in proliferative and differentiation responses but there is good evidence for a role in more chronic opioid receptor activation and possibly withdrawal [42–44]. This kinase along with p38 also plays a role in apoptosis [45]; the distinction of roles is not clear cut. In terms of activation, two pathways have been suggested-the first involving a typical G-protein mediated event and the second via an arrestin–MAPK protein scaffold. That arrestin is activated is confirmed by the BRET assay but without full concentration response curves and pertussis toxin sensitivity experiments it is not possible to delineate the relative importance of these pathways. The fact that dermorphin and N/OFQ behaved as full agonists with no striking differences in potency suggests no ligand bias as expected for naturally occurring agonists. We have used the gpI as a more intact bioassay that (1) endogenously expresses both Mu and NOP [33] and (2) links the in vitro recombinant data set to the in vivo behavioural experiments. In this preparation, N/OFQ was sensitive to the non-peptide antagonist SB-612111 but not naloxone; dermorphin was sensitive to naloxone but not SB-612111. These antagonists displayed lower potency when tested against DeNo whose concentration response curve was additively shifted when a cocktail of antagonists was used. Collectively, these results demonstrated that the biological action of DeNo in this preparation is due to the simultaneous activation of NOP and Mu receptors despite the greater loss of potency at Mu than at NOP found in recombinant systems.

*In vivo*, after spinal administration in rats dermorphin (present results) and N/OFQ [46] elicited similar antinociceptive actions. Under the same experimental conditions, DeNo mimicked the effects of dermorphin and N/OFQ being neither more potent nor more effective. This result contrasts with the extensive literature evidence suggesting that the simultaneous activation of Mu and NOP receptor elicits synergistic antinociceptive effects both in rodents [47–49] and in non-human primates [12, 16, 50, 51]. *In vitro* studies clearly demonstrated that DeNo is able to bind and activate both Mu and NOP receptor in the same range of concentrations; this may not be the case *in vivo*. A similar situation has been previously well documented in the studies of the MDAN series of compounds [52]. Under the present experimental conditions there were almost no differences between doses inducing antinociceptive effects and those disrupting animal performance on the rotarod. Further studies are needed to assess in detail any potential spinal antinociceptive actions of DeNo.

In conclusion we have created a novel bivalent Mu-NOP peptide agonist by combining dermorphin (Mu) and N/OFQ (NOP); this molecule behaves essentially as the parent compounds. Despite this promising profile, analgesic actions of DeNo are poor in the model employed here. As a mixed molecule, this ligand represents a useful addition to the non-peptides BU08028 [53, 54], the SRI-International compounds exemplified by SR16435 [54, 55] cebranopadol and the peptide [Dmt^1^]N/OFQ(1–13)-NH_2_. DeNo may be a useful tool, particularly *in vitro* for investigating the consequences of the simultaneous activation of NOP and mu receptors.
